# Knowledge and awareness of malaria and mosquito biting behaviour in selected sites within Morogoro and Dodoma regions Tanzania

**DOI:** 10.1186/s12936-016-1332-4

**Published:** 2016-05-23

**Authors:** Mary M. Mathania, Sharadhuli I. Kimera, Richard S. Silayo

**Affiliations:** Sokoine University of Agriculture, PO BOX 3019, Morogoro, Tanzania; St. John’s University of Tanzania, PO BOX 40, Dodoma, Tanzania

**Keywords:** Malaria, *Anopheles*, Insecticide-treated nets (ITNs), Tanzania, Awareness

## Abstract

**Background:**

In Tanzania there has been a downward trend in malaria prevalence partly due to use of insecticide-treated bed nets for protection against *Anopheles* mosquitoes. However, residual malaria transmission attributed to early biting behaviour of malaria vectors is being reported. Knowledge of mosquito feeding behaviour is key to improvements in control approaches. The present study aimed to assess knowledge and awareness on malaria and malaria vectors in—Morogoro and Dodoma regions of Tanzania.

**Methods:**

A cross sectional study was undertaken in selected sites in Morogoro and Dodoma Tanzania. A structured questionnaire was administered to 218 randomly selected households from each of which the head or second in/charge and the most senior primary school child were interviewed.

**Results:**

A total of 400 participants of whom 56 % were females, were recruited into the study. Their ages ranged between nine and 58 years. Among the participants, 70.7 % had primary school education and the rest attained secondary school (16.8 %), university/college (4.0 %) and not attended school at all (8.5 %). Fifteen per cent of the participants were employed, while 45.5 % were self-employed and 39.5 % were studying. Overall, 58.5 % of respondents were knowledgeable of malaria and its vector. However, 78.8 % were not aware that early mosquito bites can transmit malaria and 86.5 % said that only midnight-biting mosquito bite was responsible for malaria transmission. The majority (66 %) of respondents visited a health facility on observing malaria symptoms while 15.8 % took anti-malaria drugs without medical consultation.

**Conclusion:**

This study has shown that *Anopheles* is well known as the night-biting vector of malaria. The majority of participants were not aware of changed biting behaviour of malaria-transmitting mosquitoes and that early outdoor mosquito bite is a risk of malaria transmission. School children have shown a better understanding of malaria and its vector. Therefore, more awareness of *Anopheles* feeding behaviour is needed.

**Electronic supplementary material:**

The online version of this article (doi:10.1186/s12936-016-1332-4) contains supplementary material, which is available to authorized users.

## Background

Malaria is a major health concern in United Republic of Tanzania, it is a leading cause of inpatient and outpatient consultations. Nearly 93 % of the mainland population lives in areas where malaria is transmitted for at least 1 month per year. Although the country has been promoting the use of long-lasting insecticide-treated nets (LLINs), there are still between 60,000 and 80,000 malaria attributable deaths per year, mainly children under the age 5 years and pregnant women [1, 2]. The magnitude of the disease is more profound where knowledge and awareness of malaria and its vectors, health infrastructure and human resources for health are poor [2, 3].

Vector control is the major component of the global strategy for malaria control which aims at preventing parasite transmission mainly through interventions targeting adult *Anopheles* mosquitoes by protecting individuals who sleep or rest indoors [4, 5]. The use of pyrethroids in LLINs and indoor residual spray (IRS) has increased dramatically and become the cornerstone of malaria control programmes [4–6]. Currently the Ministry of Health and Social Welfare emphasizes a scale-up of insecticide-treated net distribution programmes under the Roll Back Malaria initiative [6]. To achieve this, the country embarked on countrywide free distribution of LLINs to make sure every household owned treated bed nets [6]. Despite the impressive success that had been achieved by use of LLINs and IRS targeting vectors that feed and rest indoor, complete elimination of malaria has rarely been achieved [7, 8]. Still there is residual malaria transmission in different part of the world described in several studies [7–11]. Many African malaria vectors, including *Anopheles gambiae* s.s, *Anopheles arabiensis* and *Anopheles funestus* have shown physiological and behavioural resistance to the insecticides applied on bed nets [12]. Physiological resistance means the expression of resistance gene in mosquito trait which can cause the mosquito to contact the insecticide but not die, i.e. knock down effect. Behavioural resistance describes any modification in mosquito behaviour (feeding and resting pattern) that helps to avoid the physical contact or lethal effects of the insecticide [7]. Beside its insecticidal activity, pyrethroids cause irritations to mosquito which leads to reduction in both the rates of house entry and successful blood feeding by those which entered in the house. Furthermore, the excito-repellent effects of insecticide influenced the mosquito to change their resting and feeding behaviour, whereas before the use of LLINs malaria vectors, especially the *An. gambiae* complex, were endophagic and bite in the middle of the night [13–15]. In many places throughout Africa a reduced indoor biting of *Anopheles* mosquitoes was reported to be due to ITNs and IRS use [13–15]. Malaria vectors have shown substantial change in feeding behaviour whereby these mosquitoes start biting early in the evening, before people have retired to bed where they could be protected by insecticide-treated bed nets [8–11, 16–18]. For this reason, LLINs can confer useful but incomplete personal protection. Studies done in Tanzania on human-mosquito interaction found that *Anopheles* biting occurred early evening where people are still active [11, 17], and this new behaviour pattern of mosquitoes may significantly increase the risk for malaria transmission. Elimination of malaria requires great focus on community knowledge, awareness and practice on malaria control strategies. Inappropriate community understanding of malaria and its vectors can draw back all effort done by malaria control programmes. Despite the enormous impact of this mosquito species behaviour, relatively little is known about community knowledge and awareness on their changed feeding behaviour.

This study was designed to assess community knowledge of malaria and malaria vector as well as awareness on changed biting behaviour of *Anopheles* mosquitoes in selected areas of Morogoro and Dodoma Tanzania. Understanding knowledge and awareness of malaria and its vectors will help communities to fight against malaria.

## Methods

### Study area

The study was conducted in two regions of Tanzania: Dodoma and Morogoro. The choice of study sites was based on prevalence of malaria and availability of permanent and seasonal breeding sites e.g. presence of rice paddies. Morogoro is among regions with high prevalence (13.0 %) of malaria compared to Dodoma (2.5 %) [2]. Morogoro town with a population of 350,000 is situated in eastern Tanzania (6°49′S and 37°40′E) at average altitude 522 m above mean sea level. The study site on lower slopes of Uluguru Mountains experiences heavy rainfall from February to June. Total average annual rainfall is 783.5 mm, mean relative humidity 72 %, minimum temperature 22 °C, and maximum temperature 33 °C during wet seasons. On the other hand Dodoma town has a population of 410,956, is located 6°25′S and 35°75′E. It has very low rainfall (total annual average rainfall 478.4 mm), mean relative humidity 67 %, minimum temperature 22 °C, maximum temperature 31 °C during wet seasons and is considered to be semi-arid. In Dodoma, rainfall peaks in February to April. This study was conducted during the dry season (July to August 2014).

### Study design and sampling procedure

The study was a cross sectional survey. A sample of 218 households was drawn from two regions by systematic random sampling. A typical household was defined as a residential house owned or rented and occupied by one or more than one family. Household head or second in-charge and senior primary school child who agreed to participate were involved in the study.

Sampled households were selected randomly whereby the first household was chosen at random, and subsequent units were obtained by calculating a sampling interval. Sampling frame was drawn from the ward executive office where list of households was obtained based on the Census 2012. A total of 218 households, 112 from Morogoro and 106 from Dodoma were involved in the study (Table 1).

**Table 1:** Description of study site and study participants

Study site	Number of households	Number of participants (n)
Dodoma	106	Adults 136
		Pupils 64
Morogoro	112	Adults 114
		Pupils 86
Total	218	400

### Data collection procedure

A structured, pre-tested Kiswahili (as translated from English version) questionnaire consisting of open-ended and closed questions was used as data collection tool. It was designed to be completed in the presence of an enumerator and collected after completion. For each household, two persons namely the head or second-in-charge and the most senior primary school child who were available during the study were selected to fill in the questionnaire. In order to understand knowledge and awareness of malaria and mosquitoes, seven questions were asked. These questions were: what causes malaria, risk of malaria transmission, name of malaria vector, what is active mosquito biting time, potential breeding site, malaria symptoms, what to do when encountering those symptoms. Questions on awareness of changed biting behaviour of *Anopheles* were focused on early mosquito bites in relation to malaria transmission. For illiterate respondents, the enumerator carried out interviews using the same questionnaire. Primary school children had an additional question (Additional file 1), which was asked to mention what health information they get more frequent at school between HIV and malaria.

### Data processing and analysis

Data collected were coded and entered into Excel then plugged into Epi Info 7 ready for data analysis. All questions on knowledge of mosquitoes and malaria, and awareness of changed biting behaviour of *Anopheles* mosquito were scored with one mark for correct answer, so as to categorize participants with ‘good knowledge’ zero was given for incorrect answers and those participants giving incorrect answers were categorized as having ‘poor knowledge’. For awareness; participants who got correct answers were categorized as being ‘aware’ and those participants got incorrect answers were categorized to be “not aware”. The average score was used as cut-off point.

All data were summarized into proportions and cross tabulations was done to look for association between independent and dependent variables. Chi square test (χ^2^) was used to test for significance and the association was considered statistically significant if p value was <0.05.

Logistic regression analysis was performed to test the association between binary response variables and their corresponding explanatory variables.

### Ethical consideration

Ethical clearance for the studies was granted by the Sokoine University of Agriculture (SUA) Directorate for Research and Postgraduate studies. Participants were briefed on details of the study and assured confidentiality, and signed written informed consent (or oral in case illiterate respondent) to participate in the study. Anonymity was assured by identifying respondents by numbers.

## Results

### Demographic characteristics of study participants

A total of 218 households were visited and agreed to participate in the study. Among these, 112 households were interviewed at the Morogoro and 106 at the Dodoma. The highest proportions (41.5 %) of participants were aged 25–45 years old. While the age group <10–17 years comprised 37.5 %, 18–24 years comprised 12.5 % and age group above 45 years comprised 8.5 % respondents. The ratio male to female was approximately 0.8 (176/224). The majority of the heads/adult second-in-charge were self-employed. Seventy percent of participants had primary school level of education (Table 2).

**Table 2:** Socio-demographic characteristics of study participants in two regions (Dodoma and Morogoro): n = 400

Variables	Dodoma n (%)	Morogoro n (%)	Both n (%)
Age group (years)			
<10–17	64 (32.0)	86 (43.0)	150 (37.5)
18–24	37 (18.5)	12 (6.0)	49 (12.5)
25–45	71 (35.5)	95 (47.5)	166 (41.5)
>45	28 (14.0)	7 (3.5)	35 (8.5)
Sex			
Male	96 (48.0)	80 (40.0)	176 (44.0)
Female	104 (52.0)	120 (60.0)	224 (56.0)
Education level			
Not attended school	34 (17.0)	0 (0)	34 (8.5)
Primary school	115 (57.5)	168 (84.0)	283 (70.7)
Secondary school	42 (21.0)	25 (12.5)	67 (16.8)
College/university	9 (4.5)	7 (3.5)	16 (4.0)
Occupation			
Employed	28 (14.0)	32 (16.0)	60 (15.0)
Self-employed	100 (50.0)	82 (41.0)	182 (45.5)
Studying	72 (36.0)	86 (43.0)	158 (39.5)

### Knowledge of malaria, malaria vector and awareness toward biting behaviour of *Anopheles* mosquitoes

Table 3 presents the respondent’s knowledge and awareness of malaria and malaria vector. In total, 250 household heads and 150 senior primary school children were interviewed during study period. It was found that majority of respondents confused causation and malaria transmission risk, only 9.0 % participants correctly mentioned plasmodium as a causative agent for malaria while the remainder said malaria is caused by mosquitoes, bacteria, worms and other factors (69.0, 9.5, 9.0, and 9.5 % respectively). Majority of respondents (86.2 %) correctly associated malaria transmission with mosquito bites. When asked to name the vector transmitting malaria, 75.5 % of respondents correctly mentioned *Anopheles* while 11.5 and 7.3 % mentioned *Culex* and *Aedes* respectively and 5.7 % indicated they did not know. With respect to perceived health problems in their households, 86.7 % of respondents mentioned malaria as number one disease, while 13.3 % of participants said typhoid, diarrhoea, UTI (Urinary Tract Infection) and fungal infection were other health problems in their family. Symptoms such as headache (38.7 %), high fever (37.3 %), and vomiting (13.4 %) were three most frequently mentioned sign and symptoms of malaria. Few participants pointed out joint pain and shivering (5.3 %) as other signs and symptoms of malaria. Sixty-six percent of respondent said they go to hospital when they encounter such symptoms while 15.8 % reported to use anti-malarial without laboratory checkup. Regular use of bed nets for prevention of malaria was mentioned by 71.0 % of the respondents, while other mentioned measures used to prevent mosquito bites were mosquito coils (12.8 %), mosquito spray (11.8 %) and other (3.4 %) (Wearing long sleeved cloths, burning local herbs).

**Table 3:** Respondent’s knowledge of malaria, malaria vector and preventive measures in two selected cities (Dodoma and Morogoro)

Variables	Dodoma n (%)	Morogoro n (%)	Both n (%)
Cause of malaria			
Mosquito	118 (59.0)	158 (79.0)	276 (69.0)
Bacteria	22 (11.0)	16 (8.0)	38 (9.5)
Worms	4 (2.0)	8 (4.0)	12 (3.0)
Plasmodium	33 (16.5)	3 (1.5)	36 (9.0)
Others	23 (11.5)	15 (7.5)	38 (9.5)
Risk of malaria transmission			
Mosquito bite	185 (92.5)	160 (80.0)	345 (86.2)
Don’t know	4 (2.0)	33 (16.5)	37 (9.3)
Others	11 (5.5)	7 (3.5)	18 (4.5)
Name of the vector			
*Anopheles*	150 (75.0)	152 (76.0)	302 (75.5)
*Culex*	26 (13.0)	20 (10.0)	46 (11.5)
*Aedes*	17 (8.5)	12 (6.0)	29 (7.3)
Don’t know	7 (3.5)	16 (8.0)	23 (5.7)
Household health problem			
Malaria	172 (86.0)	175 (87.5)	347 (86.7)
Others (typhoid, diarrhoea, UTI, fungi)		25 (12.5)	53 (13.3)
Don’t know	18 (9.0)	23 (11.5)	41 (10.2)
Malaria symptoms			
High fever	56 (28.0)	99 (49.5)	155 (38.7)
Headache	96 (48.0)	53 (26.5)	149 (37.3)
Vomiting	28 (14.0)	26 (13.0)	54 (13.4)
Others	20 (10.0)	22 (11.0)	44 (10.6)
Action taken when encounter these symptoms			
Hospital	119 (59.5)	145 (72.5)	264 (66.0)
Pain killer	37 (18.5)	25 (12.5)	62 (15.5)
Relative	4 (2.0)	2 (1.0)	6 (1.5)
Traditional healer	2 (1.0)	3 (1.5)	5 (1.2)
Use anti-malarial without checkup	38 (19.0)	25 (12.5)	63 (15.8)
Early *Anopheles* bite transmit malaria			
Yes	38 (19.0 %)	47 (23.5 %)	85 (21.2)
No	162 (81.0 %)	153 (76.5 %)	315 (78.8)
Only midnight bites responsible for malaria transmission			
Yes	166 (83.0)	179 (89.5)	345 (86.5)
No	34 (17.0)	21 (10.5)	55 (13.5)
Methods used for personal protection			
LLINS	138 (69.0)	146 (73.0)	284 (71.0)
IRS	4 (1.0)	0 (0)	4 (1.0)
Mosquito coils	30 (15)	21 (10.5)	51 (12.8)
Mosquito spray	18 (9.0)	29 (14.5)	47 (11.8)
Others	10 (5.0)	4 (2.0)	14 (3.4)
For school children			
What health information do you get more frequent at school			
HIV	48 (70.5)	75 (75.5)	123 (73.2)
Malaria	20 (29.5)	25 (25.0)	45 (26.8)

**Fig. 1:**
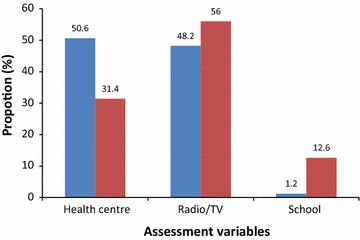
Preferred source of information on malaria and its vectors by study participants (in *Blue* adults; in *Red* school children)

With regards to awareness of biting behaviour of *Anopheles* mosquitoes, 64.5 % of study participants were not aware of changed feeding behaviour of malaria vectors. The majority of participants 78.8 % were not aware that early evening *Anopheles* bite can transmit malaria. Most 86.5 % participants believe that only midnight mosquito bites are responsible for malaria transmission. Health Centre was mentioned by adult participants as appropriate place to get health information whereas most schoolchildren pointed radio, television and school as the source of information (Fig. 1).

Furthermore, schoolchildren were asked to mention which health subject was taught regularly, Majority of pupils (73.2 %) declared that HIV/AIDS was the subject frequently taught more than malaria (Table 3).

### Correct knowledge on malaria, malaria vectors and mosquito biting risk

**Fig. 2:**
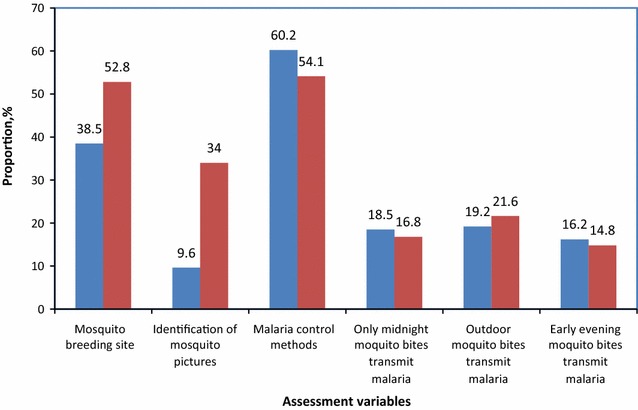
Correct knowledge on mosquito and malaria transmission risk by age category (in *Blue* adults; in *Red* school children)

**Fig. 3:**
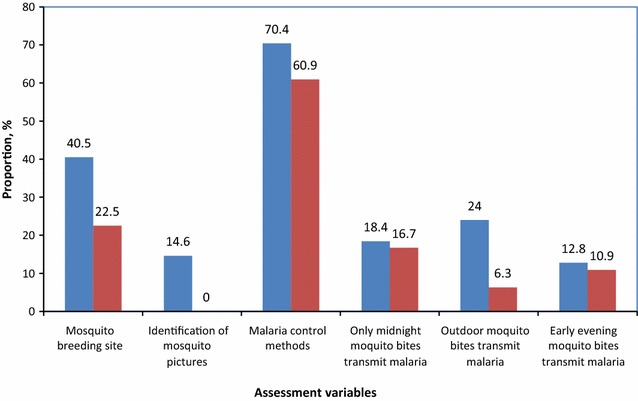
Correct knowledge on mosquito and malaria transmission risk by education level (in *Blue* literate; in *Red* illiterate)

**Fig. 4:**
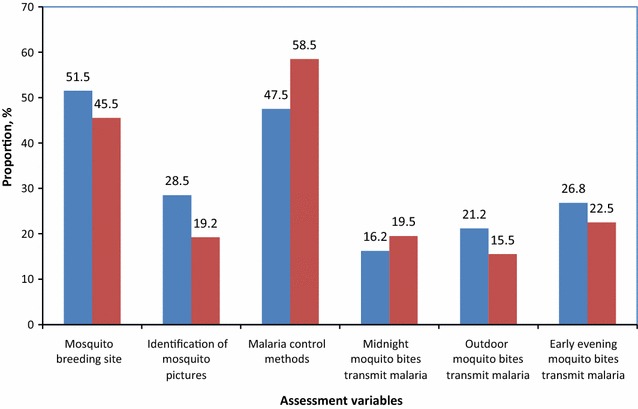
Correct knowledge on mosquito and malaria transmission risk by study location (region) (in *Blue* Dodoma; in *Red* Morogoro)

Figures 2, 3 and 4 presents different responses between binary variables based on correct knowledge on mosquito breeding sites, risk of malaria transmission, identification of mosquito pictures as well as awareness on malaria vector control methods.

### Age

The results in Fig. 2 shows that school children had more knowledge on mosquito breeding site compared to adult respondents (p = 0.000009, *χ*^*2*^ = 19.79). Moreover, more school children correctly identified adult and larval mosquito pictures than adults (p = 0.002, *χ*^*2*^ = 9.65). Similarly, majority of school children had correct knowledge on outdoor mosquitoes in relation to risk of malaria transmission. There were no significant differences on awareness of malaria control methods, correct knowledge on midnight mosquito bites, and malaria transmission risk by early evening mosquito bites between age groups (p > 0.05). Logistic regression shows that lower age is significantly associated with knowledge on mosquito breeding sites (OR = 2.46, 95 % CI 1.5–3.9, p = 0.0002) but other variables were not associated with age (Fig. 2).

### Education

Figure 3 shows that literate respondents had more knowledge of mosquito breeding sites compared to illiterate respondents (p = 0.0025, *χ*^*2*^ = 9.12). Literate respondents correctly identified adult and larval mosquito pictures than illiterates (p = 0.00056, *χ*^*2*^ = 11.8). The majority literate respondents had correct knowledge on outdoor mosquitoes in relation to risk of malaria transmission (p = 0.0016, *χ*^*2*^ = 9.9). Furthermore, the results show that awareness of malaria control and prevention methods was higher in literate than illiterate respondents (p = 0.00021, *χ*^*2*^ = 13.69). There were no significant differences between literate and illiterate respondents on correct knowledge of midnight mosquito bites, as well as knowledge of malaria transmission risk by early evening mosquito bites (p > 0.05). Logistic regression shows that education (literate) is significantly associated with awareness of malaria vector control and prevention methods (OR 3.9, 95 % CI 1.8–8.2, p = 0.0003). Other response variables were not associated with education (Fig. 3).

### Study location (Region)

The results in Fig. 4 shows that there were no significant differences between the study locations for most of response variables investigated (p > 0.05). However more Dodoma than Morogoro respondents had correct knowledge related to risk of malaria transmission by outdoor mosquitoes (p = 0.04, *χ*^*2*^ = 4.3) (Fig. 4).

## Discussion

This study investigated the knowledge and awareness on malaria and assesses the community understanding on changed feeding behaviour of *Anopheles* mosquitoes. Findings of this study indicate that malaria is a public health problem in many households. More respondents had good knowledge on malaria transmission risk, signs and symptoms. Despite the confusion observed among respondents on cause and transmission risk, majority pointed out the role of mosquito in malaria transmission by associating malaria with mosquito bite. The observed better knowledge about vector transmission risks may have been influenced by information, education and communication (IEC) facilities, which the respondents are exposed. Several studies have reported similar findings in malaria endemic areas [19–25]. Like elsewhere in Africa, the study participants identified malaria mainly on the basis of the symptoms of headache, high fever and vomiting, these findings conform to previous studies [20, 23–25]. The health centre was most preferred by adult participants, whereas more schoolchildren pointed radio, television and school as the source of information. Frequent utilization of health centre by adults in seeking health care and time spent at home by schoolchildren after school hours could explain the reason of their choice. This finding is in line with study done by Edson and Kayombo [26]. Despite better understanding of malaria transmission risk, signs and symptoms, the study participants demonstrated knowledge gap on mosquito breeding sites by stating that garbage and long grasses were potential breeding sites for malaria vector. A similar finding was reported in Pune district, India, where the majority of study participants reported garbage as breeding sites for malaria vector [27]. Many scholars have suggested source reduction and environmental management to be incorporated in malaria integrated programmes [28–31]. In the present study, participants demonstrated low level of knowledge on malaria breeding sites. Thus the public health education intervention programs should design health package which will cover existing knowledge gap as the country has started to encourage people to concentrate in source reduction and environmental management.

Interestingly, when the participants were given pictures of different mosquito species about quarter of participants correctly identified pictures of adult and larval stage of *Anopheles* mosquitoes. When further analysis was done, schoolchildren scored higher than adults. Health information given to pupils at school could explain the difference of this score. Although majority of pupils did not point out school as main source of malaria information, this study had realized that schoolchildren have better knowledge on malaria and mosquitoes than adults. Perhaps this findings could alarm malaria intervention officers to involve school teachers in malaria intervention programmes because children are enthusiastic to practice what they have learnt [26]. Since most children in Tanzania attend day school they could be a good channel for disseminating health information among household members when they go back home. What is important is to empower school teachers with appropriate health knowledge i.e. malaria through seminars and other communication means. Furthermore schoolchildren declared to be taught HIV/AIDS more than malaria, while malaria was mentioned by majority of participants to be the number one disease among top five diseases in their households. This observation would call for revision of school curriculum so that malaria and HIV/AIDS lessons could be given the same strength in primary school teachings.

Many studies have reported insecticidal pressure, which selectively caused behavioural adaptation of mosquitoes in response to indoor interventions (i.e. LLINS and IRS). This behaviour reduced the density of endophagic mosquitoes to relative more early outdoor feeding behaviour which coincides with human outdoor activities exposing people to residual malaria transmission [8–11, 16, 17, 32, 33]. The present study found that majority of participants was not aware that *Anopheles* mosquitoes can bite early evening and these bites are responsible for residual malaria transmission. This could be the outcome of the constant message carried on television and radio pointing malaria as a disease transmitted by a midnight-biting mosquito, thus more than 70 % of participants used LLINs as their personal protection against mosquito bite than other methods. The use of insecticide-treated nets (ITNs) has been reported by many studies as the major recognized method of personal protection against mosquitoes bite in malaria endemic areas [20, 22, 23, 34, 35]. Although ITN use still provides useful protection, its protective efficacy is limited to those who sleep under ITNs and exclude those who are actively outdoors. Therefore, health authorities should impart knowledge to the community about changed feeding behaviour of malaria vectors so that people can protect themselves against early outdoor mosquito bites. To fight against early outdoor bites, which cause residual malaria transmission, additional important options like mosquito sprays and coils, wearing long sleeved cloths, larviciding and environmental management can be used to complement ITNs and IRS interventions.

## Conclusion and recommendation

In this study, respondents showed moderate understanding of malaria and its vectors although knowledge and awareness of breeding sites and mosquito biting times was poor. School children have shown good understanding of malaria and its vectors; therefore there is a need to empower teachers with appropriate health information including malaria so that they can deliver malaria information to the pupils. School children are future generation so teachers—school children partnership will play a great role in malaria control strategies. Residual malaria transmission is a new challenge which is sufficiently intense across most of the tropical countries which render malaria elimination to be unfeasible, thus information, educational and communication (ICE) facilities, such as television, radio, posters, schools, health centre could be used to disseminate information of changed feeding behaviour of malaria vector so that people can protect themselves against early outdoor mosquito bite by using mosquito repellents, long sleeved cloths and environmental manipulations.

## Additional file

10.1186/s12936-016-1332-4 Household questionnaire.
