# Supplementary material for: Knowledge and awareness of malaria and mosquito biting behaviour in selected sites within Morogoro and Dodoma regions Tanzania
Source: Malar J. 2016 May 23;15:287. doi: 10.1186/s12936-016-1332-4 (PMC4877798; doi:10.1186/s12936-016-1332-4)
Supplement: Supplementary file 1 — 10.1186/s12936-016-1332-4 Household questionnaire. [file 12936_2016_1332_MOESM1_ESM.docx]

**HOUSEHOLD QUESTIONNAIRE: ENGLISH VERSION**

**KNOWLEDGE AND AWARENESS ON MALARIA, MALARIA VECTOR CONTROLS AND MOSQUITO BITING BEHAVIOR IN SELECTED SITES WITHIN MOROGORO AND DODOMA REGIONS TANZANIA**

| **S/N** | | **Variables** | **Response** | |
| --- | --- | --- | --- | --- |
|  | | **Region …………………………** | | |
|  | | **Questionnaire #** | | |
| **SOCIODEMOGRAPHIC CHARACTERISTICS** | | | | |
|  | Age | | ……. | |
|  | Sex | | 1. Male | 1. Female |
|  | Ward and street | | - 1. Ward……………. | 1. Street…………………. |
|  | Level of education | | 1. No formal education 2. Primary education 3. Secondary education | 1. College /university 2. Others (specify)…… |
|  | Marital status | | 1. Single 2. Married 3. Cohabited | 1. Divorced 2. Widowed 3. Others(specify) |
|  | Occupation status | | 1. Self employed 2. Employed | 1. Studying 2. Others (specify}…… |
| **MALARIA, MALARIA VECTOR CONTROLS AND MOSQUITOES-(KNOWLEDGE) Choose appropriate answer(s)** | | | | |
|  | What causes malaria? | | 1.Mosquito  2. Bacteria  3. Worms | 1. Plasmodium 2. Others, explain…..…… |
|  | What is a risk mode of malaria transmission? | | ……………………………………………….. | |
|  | What is the name of the mosquito which transmits malaria? | | - 1. Anopheles   2. Culex | 3. Aedes  4. Don’t know |
|  | What are common breeding sites for mosquitoes? | | 1. Garbage/trash 2. Running water | 1. Stagnant water 2. Long grasses |
|  | What is the most important health problem affecting your household | | ……………………………………………………………………………. | |
|  | Have you suffered from malaria? | | 1. Yes | 1. No |
|  | What are symptoms and signs of malaria? | | 1. High fever 2. Headache 3. Vomiting | 1. Joint pain 2. Shivering/Rigors 3. Other (specify)………. |
|  | When you get these symptoms above what do you do? | | 1. Go to hospital for check up and treatment 2. Use antimalarial without check up | 1. Go to traditional healer 2. Use pain killer 3. Other (specify)….. |
|  | What is your preferred source of information for Malaria and its vector | | 1. Health Centre 2. Radio | 1. Television 2. At school |
| **MALARIA, MALARIA VECTOR CONTROLS AND MOSQUITOES -(Awareness) Choose one correct answer** | | | | |
|  | What time can malaria vector start biting? | | 1. Midnight 2. Early evening | 1. Anytime |
|  | Is midnight mosquito only responsible for malaria transmission? | | 1. Yes | 1. No |
|  | Can early Anopheles bite transmit malaria? | | 1. Yes | 1. No |
|  | Can outdoor mosquito bite transmit malaria? | | 1. Yes | 2.No |
|  | What convenient protection methods against mosquito bite do use in your household? | | 1. LLINs 2. IRS 3. Mosquito spray | 1. Mosquito coils 2. Others (specify)….. |
|  | In your household do you own mosquito nets? | | 1. Yes | 1. No |
|  | Who sleeps under mosquito nets? | | 1. Pregnant mother 2. Mothers and under-five children | 1. Father only 2. All people in the house 3. Others (specify) |
|  | Where did you get this net? | | 1. Mass campaign 2. Retail shop | 1. Health facility 2. Others(specify)……. |
| **UELEWA JUU YA MBU ANAYEENEZA MALARIA.**  **Put tick (√) to the pictures of mosquito which transmit malaria, (X ) to pictures of mosquito which do not transmit malaria and (0) if you don’t know** | | | | |
| 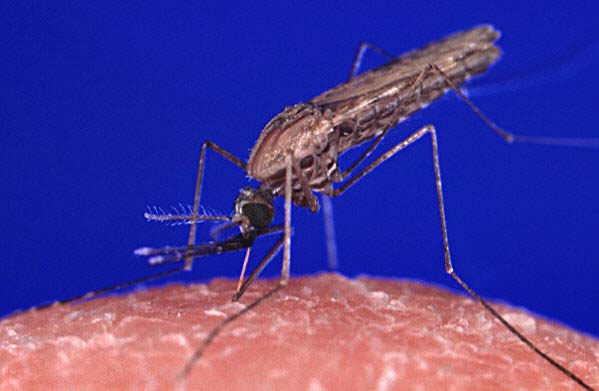  i)………………………………… | | | 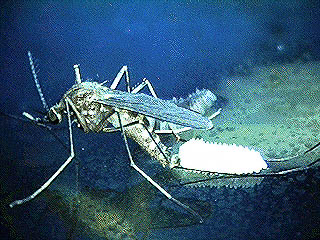  ii)……………………………….. | |
| 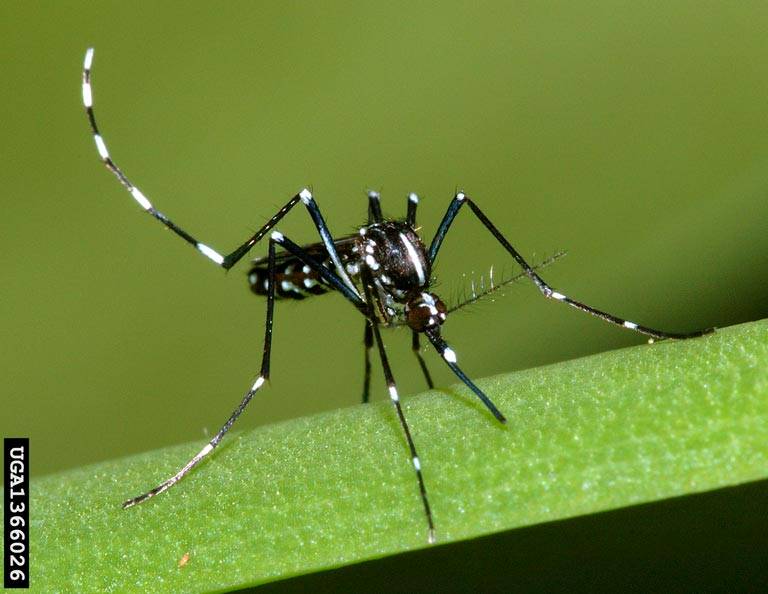  iii)………………………………… | | | 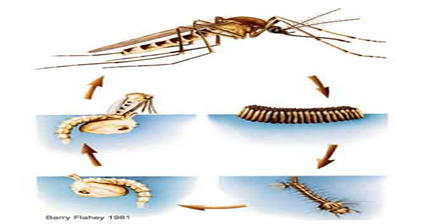  iv) ………………………… | |
| 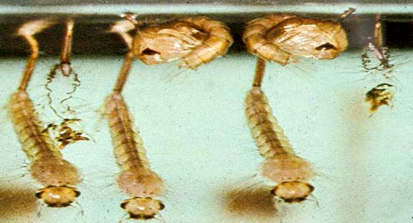  v)…………………………………… | | | 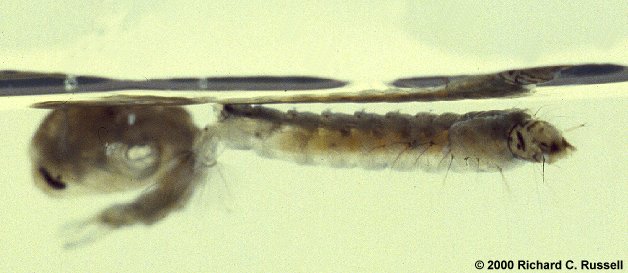  vi)…………………………………… | |
| **FOR PUPILS ONLY** | | | | |
|  | What health information do you get more frequent at school | | 1. HIV ` | 1. Malaria |

***Thank you for your participation!***

**SWAHILI VERSION**

**DODOSO LA KUPIMA UELEWA JUU YA MALARIA, MBU WANGATAO MAPEMA/JION NA NJIA ZA KUJIKINGA NA MBU HAO**

| **S/N** | | **Maelezo** | | **Majibu** | | |
| --- | --- | --- | --- | --- | --- | --- |
| **1.** | | Mkoa…………………………………………….. | | | | |
| **2.** | | Namba ya dodoso | | | | |
| **Taarifa ya mshiriki** | | | | | | |
|  | | Umri (Miaka) | |  | | |
|  | | Jinsia | | 1.Me | | 2.Ke |
|  | | Kata na Mtaa unamoishi | | Kata …………………………….. | | Mtaa …………………………… |
|  | | Kiwango cha elimu | | 1.Sijasoma  2.Elimu ya msingi | | 3.Elimu ya sekondari  4.Chuo/ Chuo kikuu  5.Majibu mengine…………… |
|  | | Hali ya ndoa | | 1.Sijaowa /Sijaolewa  2.Nimeowa /Nimeolewa  3.Tunaishi bila ndoa | | 1. Tumeachana 2. Nimefiwa mke/mme 3. Mengineyo... |
|  | | Ajira | | 1.Nimeajiajiri   - 1. Nimeajiriwa | | 1. Nasoma 2. Menyineyo... |
| **Elimu juu ya malaria, mbu na njia za kujikinga dhidi ya malaria (Chagua jibu au majibu sahihi)** | | | | | | |
|  | | Ugonjwa wa malaria unasababishwa na nini? | | - 1. Mbu   2. Bakteria   3. Minyoo | 4.Plasmodiam  5. Fangasi  6.Nyingine (elezea)…………… | |
|  | | Ni njia gani hatarishi inayoweza kusambaza malaria? | | ………………………………………….. | | |
|  | | Taja jina la mbu anayesambaza ugonjwa wa malaria | | 1. Anofelesi 2. Kiuleksi | 1. Aidesi 2. Sijui | |
|  | | Ni mazingira gani yanasababisha mbu kuzaliana? | | 1. Sehemu ya kutunza takataka 2. Maji yanayotiririka | 1. Maji yaliyotuama 2. Majani marefu | |
|  | | Je ni magonjwa gani yanasumbua sana familia yako? | | ………………………………………………………………………… | | |
|  | | Umeshawahi kuugua malaria | | 1.Ndio | | 2.Hapana |
|  | | Taja dalili za malaria | | 1.Homa kali  2. Kichwa kuuma  3.Kutapika | | 4.Kutetemeka  5.Maumivu ya viungo  6.Nyingine (elezea) …………….. |
|  | | Ukiona dalili hizi za malaria anafanya nini? | | 1.Ninaenda hospitali  2.Nakunywa dawa za malaria bila kupima  3.Ananipeleke kwa mganga wa jadi | | 4.Ananipa dawa za kupunguza maumivu  5. Majibu mengine…………… |
|  | | Chagua sehemu ungependa ikupatie taarifa kuhusu malaria na mbu | | 1. Kituo cha afya 2. Radio | | 1. Kwenye Runinga 2. Shuleni |
| **Mtazamo juu ya malaria, mbu na njia za kujikinga dhidi ya malaria (Chagua jibu au majibu sahihi)** | | | | | | |
|  | Muda gani ambao mbu wanaoambukiza malaria wanaanza kungata watu | | 1.Usiku wa manane  2. Mapema jioni | | | 3.Muda wowote |
|  | Mbu wanaonang’ata usiku wa manane ndio mbu pekee wanaoasambaza malaria? | | 1. Ndio | | | 1. Hapana |
|  | Mbu wanaong’ata mapema jioni wanaweza kusambaza malaria | | 1. Ndio | | | 1. Hapana |
|  | Mbu wanaong’ata nje ya nyumba wanaweza kuambukiza malaria? | | 1.Ndio | | | 2.Hapana |
|  | Njia zipi rahis unazotumia nyumban kujikinga na mbu wanaoasambaza malaria? | | 1. Chandarua kilichowekewa dawa ya muda mrefu 2. Dawa za kunyunyiza kwenye ukuta 3. Dawa za kupuliza | | | 1. Dawa za mbu za kuchoma 2. Majibu mengine, (elezea)……………………… |
|  | | Je kwenye familia yako mnamiliki chandarua? | 1. Ndio | | | 1. Hapana |
|  | | Je vyandarua hivyo mnavitoa wapi ? | 1. Kwenye kampeni za kugawa vyandarua 2. Tunanunua dukani | | | 1. Tunapewa hospitali 2. Majibu mengine, (elezea)……………………… |
|  | | Nani analala kwenye chandarua? | 1. Mama mjamzito 2. Mama na watoto chini ya miaka mitano | | | 1. Baba 2. Watu wote 3. Majibu mengine, (elezea)……………………… |
| ***UELEWA JUU YA MBU ANAYEENEZA MALARIA.***  **Weka alama ya tiki (√) kwenye picha za mbu wanaoeneza malaria, alama (X)atika picha ya mbu wasioeneza malaria na alama (0 ) kama haujuiako**   \| 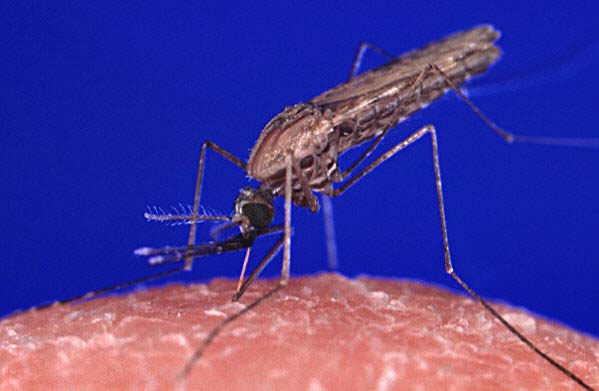  i)………………………………… \| 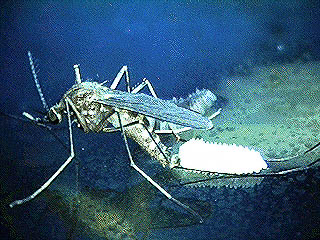  ii)……………………………….. \| \| --- \| --- \| \| 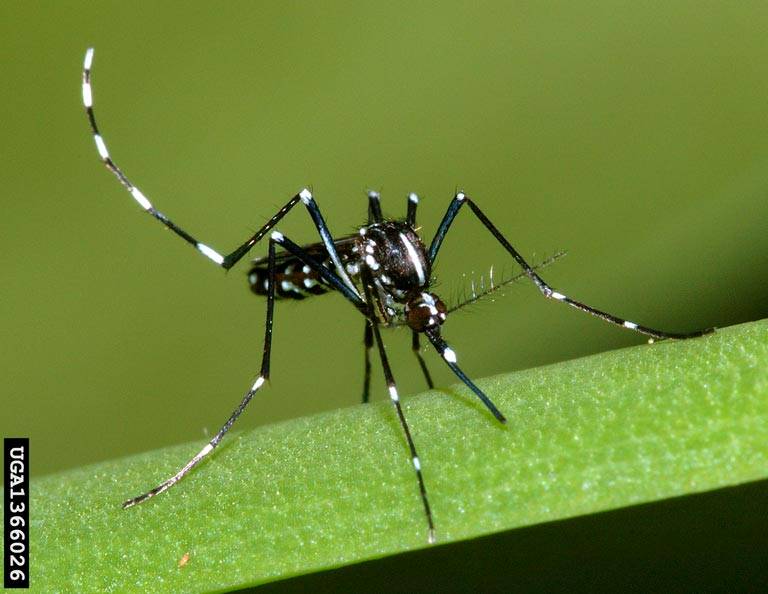  iii)………………………………… \| 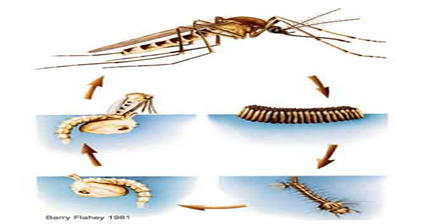  iv) ………………………… \| \| 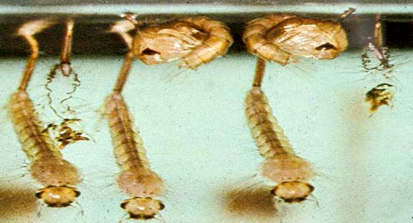  v)…………………………………… \| 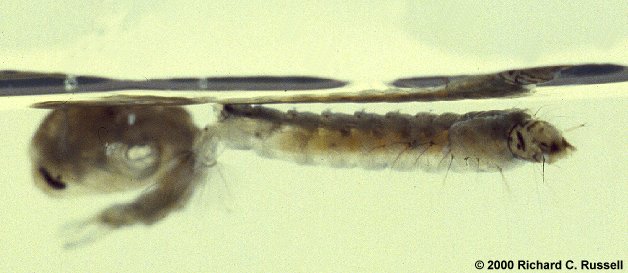  vi)…………………………………… \|   **WANAFUNZI TU** | | | | | | |
|  | | Ni habari zipi mnaazofundishwa zaidi pewa shuleni | 1. Virusi vya ukimwi na ukimwi. | | | 1. Malaria |

***Asante kwa ushiriki !***
